# Review of video-assisted thoracoscopy in children

**DOI:** 10.4103/0972-9941.58498

**Published:** 2009

**Authors:** S N Oak, S V Parelkar, K V SatishKumar, R Pathak, B H Ramesh, S Sudhir, M Keshav

**Affiliations:** Department of Pediatric Surgery, TNMC and BYL Nair Hospital, Mumbai Central, Mumbai, India

**Keywords:** Empyema, thoracoscopy, video-assisted thoracoscopic surgery

## Abstract

Open thoracotomy is the standard procedure for various thoracic diseases against which other procedures are compared. Currently Video Assisted Thoracoscopic Surgery (VATS) has gained widespread acceptance in the management of a variety of thoracic disorders. It decreases the morbidity and duration of hospital stay. A total of 133 children with various thoracic diseases who presented at a University Teaching Hospital in the Department of Pediatric Surgery, from June 2000 to December 2007, were included. Of the 133 patients, 116 patients had empyema, all of whom were subjected to VATS, and an attempt at debridement/decortication and drainage was made. Other thoracic disorders treated included lung abscesses, lung biopsies, hydatid cysts, and so on. Patients with empyema were treated according to their stage of disease. Of the 116 patients who underwent thoracoscopy, 16 had to be converted to open surgery due to various reasons. The mean duration for removal of drain was three days and the average total duration of hospital stay was six days. Similarly the application of VATS was advantageous in other thoracic diseases.

## INTRODUCTION

The management option for empyema depends on the phase of the disease. Antibiotics and intercostal drainage (ICD) is the mainstay of the treatment, but some cases may need decortication. During the early (exudative) phase of empyema, the fluid is thin and ICD may suff ice, but with progression of the disease into the second (fibrinopurulent) phase, the fluid becomes gelatinous with fibrin strands, loculations, and adhesions, in which case, debridement and decortication become necessary. The last phase (organized) is characterized by an increased cross-linking of the fibrin, trapping bacteria in the interstices, as the fluid reabsorbs and decortication becomes essential.

Video-assisted techniques offer distinct advantages in the accurate staging of the disease process, effectiveness of management of organizing pleural disease, postoperative patient comfort and cosmesis. With thoracoscopy, the essentials of open thoracotomy and debridement can be achieved under vision, albeit with less trauma and better cosmesis.

### Aims and objectives

1. To demonstrate the usefulness of early thoracoscopy for empyema, where initial ICD is avoided.
2. To demonstrate advantages of thoracoscopy over thoracotomy in other (non-empyema) thoracic diseases.

Inclusion criteria (for empyema)

1. Duration of disease less than three weeks

Exclusion criteria (for empyema)

1. Duration of disease more than three weeks
2. Bilateral disease
3. Computerized Tomography (CT) scan showing definite evidence of bronchopleural fistula

## MATERIALS AND METHODS

A prospective study was carried out over a period of eight years (2000-2007) involving 133 patients majority of whom were cases of empyema (116). Other cases included lung abscess, parenchymal lesions, lung cysts, and so on [Table 1]. Of these cases, 69 were male while 64 were female.

**Table 1:** Procedures done by in the present study

Procedure	No. of patients	Conversion	Success rate (%)
Decortication	116	16	87
Lung biopsy	6	1	84
Lung abscess	4	0	100
Foreign body lung	1	1	0
Mediastinal lymph nodes	1	0	100
Lung hydatid*	5	5	100
Total	133	23	83

* Followed by minithoracotomy to deliver the cysts in two patients

### Empyema

A total of 116 cases of empyema were treated in various stages with thoracoscopy. Children with ages between six months and 12 years (Mean age of 3.9 years) underwent VATS decortication. There were 60 males and 56 females. Mean duration of disease prior to surgery was 10 days.

Diagnosis of empyema thoracis was based on demon stration of frank pus on pleural fluid aspiration, prior to admission.

All these patients were evaluated with chest skiagrams, pleural fluid analysis, and CT thorax (P 1 C), to adequately visualize the extent of the disease, loculated collections, thickened pleural peel, shift of the mediastinum, and status of the contralateral lung. Findings of loculations, thickened pleura, and collapsed lung suggested empyema.

Depending on the stage of the disease and the duration of the symptoms, a decision was taken as to the type of surgery to be performed and the necessity of ICD insertion. The child would then be posted for VATS on the next available day as per the list (2-5 days). Patients who were not in distress were posted for primary VATS and debridement (50 cases).

All patients were operated under general anesthesia and all patients received parenteral antibiotics postoperatively until the removal of the ICD.

#### Thoracoscopy

Thoracoscopy was performed under general anesthesia, with dual lung ventilation, and with the patient in a lateral position. Two 5 mm ports were used in all patients. A primary port was placed through a prior ICD site or in case of primary VATS in the fifth or sixth intercostal space (ICS) in the mid-clavicular line, through which a zero degree wide angle scope was introduced. The CO_2_ insufflation pressure was maintained between 4-6 mm Hg at a flow rate of 1 liter/minute. The placement of a second port was based on thoracoscopic findings. Initial dissection and creation of space was achieved by CO_2_ insufflation and release of adhesions with the tip of the scope or tip of the suction cannula under vision.

The fluid was sucked out and the peel was removed with a Maryland dissector. Sometimes long open surgical instruments passed directly through the ports and aided in the removal of the peel. An ICD was placed through the camera port before closure of the ports.

Postoperatively the patients were monitored in the recovery ward for 6-12 hours. All the patients received chest physiotherapy and breathing exercises.

An immediate postoperative radiograph was carried out, to visualize the condition of the ipsilateral and contralateral lung.

The chest tube was removed after three to four days, as soon as it stopped draining any purulent material or the ICD column stopped moving. Parenteral antibiotics were given during the hospital stay and later switched on to oral antibiotics on discharge, which was continued till resolution of the consolidation.

Adequate pain management prevented splinting and more effective breathing, thereby preventing postoperative pneumonias and other pulmonary complications.

Adequate chest physiotherapy in the postoperative period led to early chest expansion.

## RESULTS

### Video-assisted thoracoscopic surgery decortication

**Figure 1:**
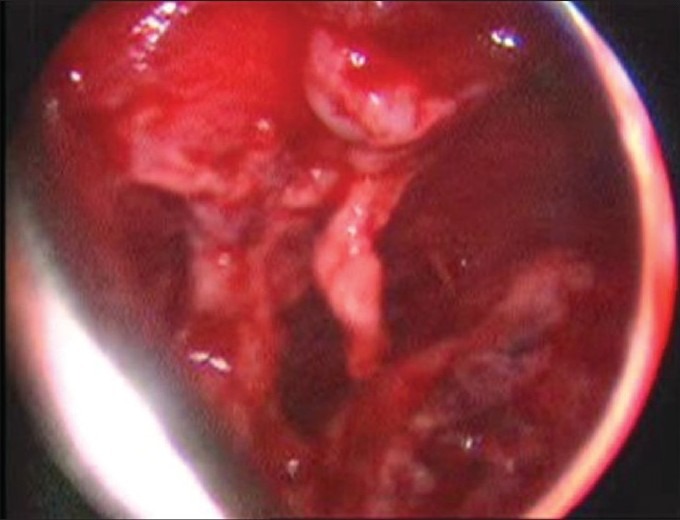
Thick peel visible on thoracoscopy

Of the 116 children with empyema, 50 (43%) of them were managed without a preliminary ICD (Primary VATS). In the cases who had presented early (5-7 days of diagnosing of empyema) toileting of the pleural cavity was done by first clearing the pleural cavity of the collection and then removing all the adhesions between the lung and the parietal pleura without a formal decortication. In patients who presented later, the pleural peel appeared to be thick and densely adherent to the underlying lung as well as to the thoracic wall and diaphragm and these needed decortications [Figure 1]. The mean operating time was 90 minutes (ranging between 150 and 45 minutes). In all patients, the intercostal drainage tubes were kept postoperatively

Postoperatively 90% of the patients showed good lung expansion on chest X-ray.

Sixteen cases (13.7%) required conversion to open procedure, due to major air leaks in 10 patients, and thick peel that resisted removal by VATS in six patients. Patients who had minor air leaks were managed conservatively.

Postoperatively the ICD was removed on an average of 3.5 days, ranging from two to five days for patients undergoing primary VATS and 2-15 days for those who had undergone VATS after a preliminary ICD. Children who required delayed ICD removal had prolonged air leaks and responded to conservative treatment.

Histopathology was obtained in all patients. Thirteen patients had biopsies confirmative of tuberculosis (11%) and in the remaining 103 patients it was reported as empyema.

Follow-up was performed at two weeks, three months, and six months.

The average duration of follow-up of patients was 10 months, follow-up radiographs becoming normal by three to four months.

One patient died in the postoperative period. The patient had excessive bleeding in the immediate postoperative period. The patient was re-explored by open thoracotomy, but no active bleeding was found, however, the patient died later, probably due to disseminated intravascular coagulation (DIC), secondary to sepsis.

Complications occurred in five patients. One patient developed pneumothorax immediately after removal of the ICD, but responded promptly to re-insertion of the ICD.

Three patients developed pneumothorax after removal of ICD one-and-a-half months, two months, and four months after VATS decortication and all of them responded to insertion of the ICD; the pneumothorax might have been due to recurrent or continued infection.

One patient developed hydropneumothorax one month after discharge and needed an open procedure and lobectomy of the involved lobe. Biopsy was reported as tuberculosis.

### Video-assisted thoracoscopic surgery in hydatid cyst of lung

**Figure 2:**
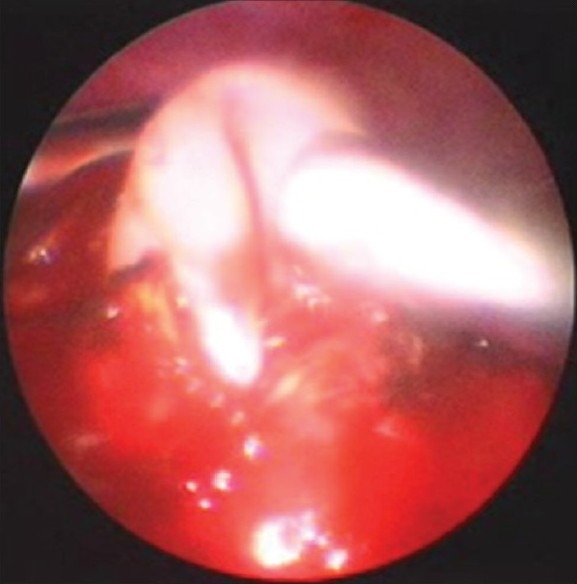
Hydatid cyst being removed from left lung

Four children with hydatid cyst of the lung were treated thoracoscopically. One patient had two cysts in the lower lobe. Three children were managed thoracoscopically [Figure 2] with a minithoracotomy for closure of air leaks. The other patient underwent VATS-assisted minimal resection of the involved lobe.

### Video-assisted thoracoscopic surgery in trauma

**Figure 3:**
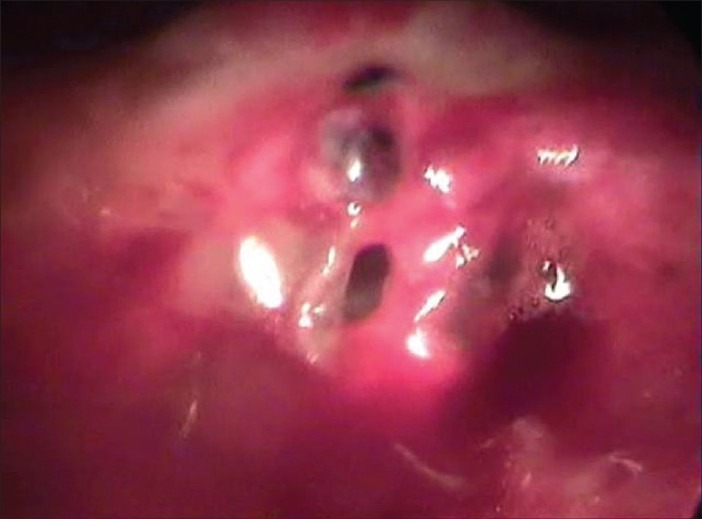
Traumatic bronchial tear visualized on thoracoscopy

We had a six-year-old boy who presented with a blunt injury to the chest. A chest X-ray revealed right pneumothorax. In spite of three days of intercostal drainage there was no improvement. On VATS, we found the tip of the endotracheal suction tube introduced by the anesthetist in the pleural cavity through a tear in the bronchus [Figure 3]. Thoracotomy revealed complete transection of the right main bronchus, which was repaired. The child had an uneventful recovery and is doing well.

### Video-assisted thoracoscopic surgery lung biopsy

**Figure 4:**
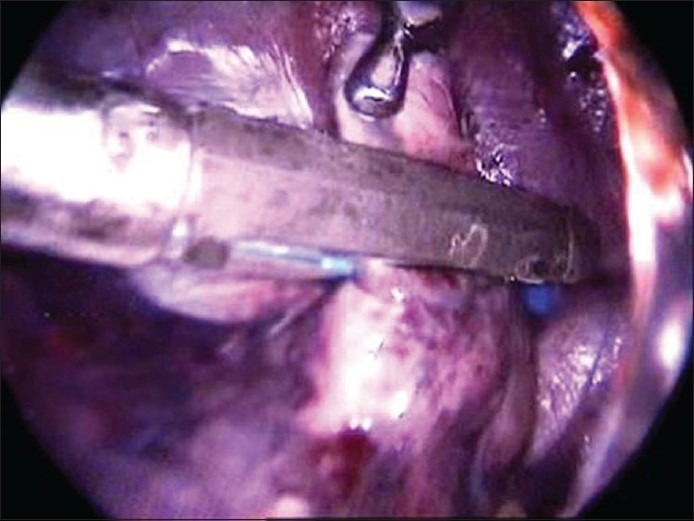
Video-assisted thoracoscopic surgery Lung biopsy using endostapler

We have done VATS lung biopsy [Figure 4] in seven cases. In three cases an endoscopic stapler was used and in the other four three cases the vicryl loop was used. We found it to be a procedure with least morbidity. Histopathology revealed tuberculosis in three cases, sarcoidosis in one case, and interstitial lung disease in three cases. One patient had dense adhesions and an open thoracotomy, and excision biopsy was done. Histopathology was reported as a pseudotumor of the lung and the patient recovered well.

### Video-assisted thoracoscopic surgery in lung abscess

In our series of four cases of lung abscess not responding to parenteral antibiotics (.2 weeks), VATS aspiration was done. All the patients responded very well to antibiotics after aspiration.

## DISCUSSION

### Video-assisted thoracoscopic surgery

Video-assisted thoracoscopic surgery (VATS) can be used to diagnose and treat various diseases of the thorax. Common indications being empyema, lung hydatid, indeterminate pulmonary nodule, lung biopsy in interstitial lung disease, mediastinal lymph node biopsy, congenital diaphragmatic hernia (CDH) repair, and so on.

#### Video-assisted thoracoscopic surgery in empyema

Empyema is a dynamic process that progresses through three stages. Stage 1, the early exudative phase, involves a collection of thin reactive fluid and few cells in the pleural space. Stage 2 is the fibropurulent phase, with large quantities of white cells and fibrin deposition, which results in the formation of loculations. Stage 3 is the organizing phase, in which a thick fibrinous peel encases the lung, limiting its mobility. The treatment of empyema is directed toward the control of infection in the pleural cavity and prevention of late pulmonary restriction, due to encasement of the lung. Although drainage with ICD (Intercostal drainage) and antibiotics may be adequate for the stage 1 disease, the presence of loculations and fibrinous adhesions may limit the success of this therapy.[1] Reports suggest that 36 to 65% of the patients are not cured by simple tube drainage.[2,3]

Kern and Rodgers introduced thoracoscopic debridement for management of children with empyema, in 1993.[4]

Thoracoscopy may be used as an early first-line in children with fibrinopurulent empyema. VATS is more effective than tube drainage and less invasive than open thoracotomy.[5–7] Various reports have also demonstrated that video-assisted thoracoscopic debridement (VATD) may cause less pain and shorten the length of stay (LOS) compared to the prolonged tube thoracotomy drain (TTD) or thoracotomy.[8–11]

According to Wait *et al*., in a randomized controlled trial, VATS was found to be more effective that ICD drainage or fibrinolytic therapy in empyema.[12]

In our 116 cases the success rate in obtaining a cure was 87% with 16 conversions (13%). Even in patients who underwent open procedure, it could be accomplished through minithoracotomy in a substantial number of patients. In a study by Shi-Ping Luh *et al*., of 234 patients with empyema in adults treated by VATS only 6.8% required conversion to open procedure.[13]

**Graph 1:**
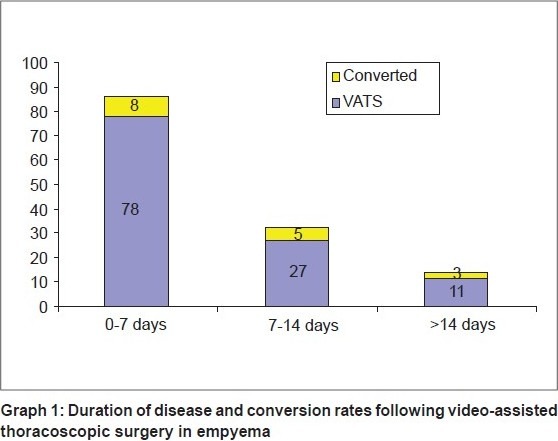
Duration of disease and conversion rates following video-assisted thoracoscopic surgery in empyema

The conversion rate was higher in our study compared to other studies [Table 2], but we found that success was better in patients with earlier intervention or in the earlier course of the disease [Graph 1].

**Table 2:** Comparison of various studies for in empyema

Study group	Duration of disease	Mean days to intercostal drainage removal	Conversion (%)
Grewal *et al*.,[18]	7.1 days	3.2 days	4
Luh *et al*.,[12] Adult population	-	4	13.7
Jeffrey R. Avansino[19]	11.2	4	2.8
Present study (116)	10	3.5	13

In 50 patients treated by primary VATS (patients with early stage disease), conversion rate was less, 4/50 patients, as compared to other patients in whom the conversion rate was higher (18%) [Graph 1].

The time up to the removal of the ICD was significantly less in patients who underwent VATS as a primary procedure, ranging from two to five days, with a mean duration to removal of ICD of 3.5 days, as compared to patients with ICD prior to VATS in whom the time up to ICD removal was significantly prolonged ranging from 2-15 days [Table 3]. Schultz *et al*., also showed a significantly shorter LOS in their study.[14]

**Table 3:** Length of stay based on preoperative history

Procedure	No. of pts	Mean duration of disease (days)	Total intercostal drainage days	Conversion
Primary Decortication	50	9	2-5	4
Following ICD	66	16	2-15	12

The success of fibrinolytic therapy as an adjunct to drainage has been reported by a number of studies.[6,15–16] However, it is felt that the use of fibrinolytic therapy should be confined to the initial three- to five-day period after chest tube placement, as it may prevent, but should not further delay, the need for surgical drainage.

Fibrinolytic therapy would have a limited value for patients with multiloculated parapneumonic effusion or empyema. Fibrinolytic agents have been reported to have adverse effects such as anaphylaxis, hemorrhage, and pulmonary edema. However, the technical and instrumental improvement associated with VATS has made it much safer and less invasive.

Chances of conversion are also more if the disease process is more than seven days, and increases as the duration increases. The importance of the application of VATS early in the course of the disease for better outcome has been demonstrated by various studies.[17] The failure rate is higher in cases presenting late [Table 4] and it is not suitable for advanced organized empyema.[18]

**Table 4:** Characteristics of failed and successful Video-assisted thoracoscopic surgery

	Completed by video-assisted thoracoscopic surgery	Converted to thoracotomy
Age	3.9 yrs	4.2 yrs
Duration of symptoms	8 days	13 days
Pre op ICD	3 days	7 days
Post op ICD	3.5 days	5 days
Post op stay	4 days	6 days
Total stay	7 days	12 days

## CONCLUSION

VATS is a safe and effective procedure for treatment of complicated parapneumonic effusion or empyema with less postoperative pain, a shorter hospital stay, and a better cosmetic result. Application of VATS early, in cases of complicated parapneumonic effusion or empyema can alter the course of the disease and give better results. The success of VATS in thoracic diseases other than empyema is comparable to an open procedure.
